# Bacteriology

**Published:** 1905-02-18

**Authors:** 


### BACTERIOLOGY.

Tuberculosis.—Much work has been done recently in relation to the life history and differentiation of the tubercle bacillus.[1] Székeley concludes that the human strain of tubercle bacillus can be transmitted to cattle and that failure is due to diminished virulence of different strains or of old cultures. This diminution of virulence is also met with in bovine tubercle bacilli. He regards the statement that bovine bacilli are harmless to man as untenable, and it is not borne out by the investigations of the British Commission on Tuberculosis of 1904, or by the experiments of Baumgarten. Theobald Smith[2] also claims to have isolated bovine tubercle bacilli from three primary cases of abdominal tuberculosis without pulmonary lesions. The cultures from these cases were characterised by the fact that the final reaction of standardised glycerine bouillon was neutral, feebly alkaline, or feebly acid, whilst that of human tubercle bacilli was strongly acid. Ravenel, too, has isolated bovine bacilli from four cases of tabes mesenterica; de Schweinitz from two; Febiger and Jensen from three; Westenhöffer from one; and the German Tuberculosis Commission from four. Heller found from 21 to 25 per cent. of primary abdominal tuberculosis at post-mortems at Kiel, a district in which the boiling or pasteurising of milk is little practised. Liguieres[3] states that the bacilli of avian tuberculosis are quite distinct from those attacking human beings, and that these again are distinct from bovine tubercle. Whilst he maintains this and states that in his experience the inoculation of human tubercle bacilli into cattle only gives rise to a transient local reaction yet he admits that sometimes the bovine bacillus may be found in man and may give rise to abdominal tuberculosis. Maher[4] of New Haven claims to have obtained from the refuse in a dark barn a bacillus ten or twelve times as long as the tubercle bacillus which was capable of passing through a cycle of changes and which eventually took on the forms and characteristics of the tubercle bacillus and was pathogenic to guinea-pigs. This bacillus he calls the bacillus maternus. It is acid-fast and can be grown best in milk made alkaline by ammonia. There are, however, so many acid-fast bacilli of this character that his results must be received with caution. Sanfelice[5] has recently described three varieties of acid-fast streptothrix occurring in air and two of these when inoculated gave rise to pseudo-tuberculous lesions.

Tuberculin as a Diagnostic Agent.—Bandelier[6] points out that one of the chief values of tuberculin as a diagnostic agent is that it distinguishes between a healed phthisis and a quiescent but still potent disease, for whereas there is a good reaction with the latter, with the former there is no reaction. Ill effects from the injections are extremely rare, but in order to secure trustworthy results certain precautions must be taken. By two-hourly observations it must be determined that there is no fever prior to the administration of the tuberculin; the injection should be given in the morning lest a rise of temperature occurring during sleep be overlooked. The cumulative reaction is that destinctive of tuberculin. The preliminary dose should be 1 mg.; the second dose 5 mg., and the third 10 mg. Larger doses may cause reaction in non-tuberculous subjects. Sometimes a reaction is obtained only after the second injection of 10 mg. Of 500 patients 34·6 per cent. reacted to 1 mg.; 31·2 reacted to 5 mg.; 19·6 per cent. reacted to the first injection of 10 mg., and 7·2 to the second. Of 7·4 per cent. of the cases the history indicated that the disease was cured and these did not react at all. Many cases, too, on discharge from the sanatorium gave no tuberculin reaction, thus pointing to their complete cure. Of eight patients who did not react to 10 mg. of tuberculin, four reacted weakly to 20 mg., and two did not react even to 50 mg. Moeller,[7] at the Berlin Brandenburg Sanatorium, has been using a tuberculin (an emulsion of tubercle bacilli) for curative purposes. His initial dose was 1-10th mg., and this was increased slowly so as to avoid a strong reaction. Of 772 patients treated with ordinary sanatorium methods, 11 per cent. were cured; of 193 cases treated with tuberculin 30·1 per cent. were cured; there were also a far higher percentage of persons relieved by the tuberculin treatment, and the deaths under this treatment were far less numerous.

Rheumatic Diseases.—Menzer[8] records a series of 47 cases of acute rheumatism treated by his anti-rheumatic serum. Of these cases he regards 43 as complete cures, one as a partial cure, and three were left with some slight impairment of function.

The average length of treatment was 53 days. He claims particular success with chronic cases that have followed acute attacks, and obstinate cases of chorea following rheumatic fever have also benefited. Relapse was seldom seen, although the majority of cases were kept under observation for a year. Of 47 acute cases 23 presented symptoms of endocarditis and in 15 cases an absolute cure was obtained, while six showed cardiac bruits without functional disturbance. As the first effect of the serum is to intensify the inflammatory process, the serum should not be used at the height of an attack of pleurisy or pericarditis or with marked mitral stenosis. In acute cases the dose of the serum is 5 c.c. In chronic cases 5 c.c. are injected every second or third day until 30 c.c. have been administered. A pause of one or two weeks is then made and the treatment recommenced later. Salicylates must not be given during the time of the serum treatment. Gibson[9] mentions a case of rheumatic pericarditis, pleurisy, and peritonitis, in which the diplococcus rheumaticus was found in the pericardial fluid. He says that, so far as is known to him, the diplococcus has never been obtained from the pleural exudates, although it is readily to be found in the stained centrifugalised urinary sediment. Possibly much of the pleurisy in acute rheumatism is secondary to heart failure.

Hydrocele Fluid.—Bargon and Cade[10] who some time ago brought forward evidence to show that essential hydroceles were caused by the rupture of minute cysts of the testis and epididymis into the vaginal sac and who showed that hydrocele fluid generally contained spermatozoa have now examined a fresh series of essential hydroceles and found spermatozoa in 52 per cent. of the cases. No spermatozoa were found in symptomatic hydroceles. In 17 cases of symptomatic hydrocele the fluid was inoculated into guinea-pigs to detect the presence of tubercle bacilli. In only one case (a spermatic cyst) was a positive result arrived at. He states that of 17 cases examined by Ravant, Lemière, Widal, and Courmont, all gave negative results.

[1] Scottish Med. Jour., Nov. 1904. [2] Amer. Jour. Med. Sc., Aug. 1904. [3] Cent. fur. Bak., Bd. 14, 1904. [4] Med. Rec., Sept. 10, 1904. [5] Rif. Med, June 4, 1904. [6] Beit. Zur. Klin. der Tuberk., Bd. ii., Hft. 4. [7] Zeit fur Tub. und Heil. Bd. v., Hft. 5. [8] Münch. Med. Woch, 1904, No. 33. [9] Brit. Med. Jour., Jan. 7, 1905. [10] Arch. Gen. de Méd., Sept. 1, 1903.

